# Precision medicine in acute lymphoblastic leukemia

**DOI:** 10.1007/s11684-020-0759-8

**Published:** 2020-10-19

**Authors:** Ching-Hon Pui

**Affiliations:** Departments of Oncology and Pathology, St. Jude Children’s Research Hospital, Memphis, TN 38105, USA

**Keywords:** acute lymphoblastic leukemia, molecular therapeutics, targeted therapy, tyrosine kinase inhibitors, immunotherapy, CAR T-cell therapy

## Abstract

The cure rate of childhood acute lymphoblastic leukemia (ALL) has exceeded 90% in some contemporary clinical trials. However, the dose intensity of conventional chemotherapy has been pushed to its limit. Further improvement in outcome will need to rely more heavily on molecular therapeutic as well as immunoand cellular-therapy approaches together with precise risk stratification. Children with *ETV6-RUNX1* or hyperdiploid > 50 ALL who achieve negative minimal residual disease during early remission induction are suitable candidates for reduction in treatment. Patients with Philadelphia chromosome (Ph)-positive or Ph-like ALL with ABL-class fusion should be treated with dasatinib. BH3 profiling and other preclinical methods have identified several high-risk subtypes, such as hypodiplod, early T-cell precursor, immature T-cell, *KMT2A*-rearranged, Ph-positive and *TCF-HLF*-positive ALL, that may respond to BCL-2 inhibitor venetoclax. There are other fusions or mutations that may serve as putative targets, but effective targeted therapy has yet to be established. For other high-risk patients or poor early treatment responders who do not have targetable genetic lesions, current approaches that offer hope include blinatumomab, inotuzumab and CAR-T cell therapy for B-ALL, and daratumumab and nelarabine for T-ALL. With the expanding therapeutic armamentarium, we should start focus on rational combinations of targeted therapy with non-overlapping toxicities.

## Introduction

**Fig. 1:**
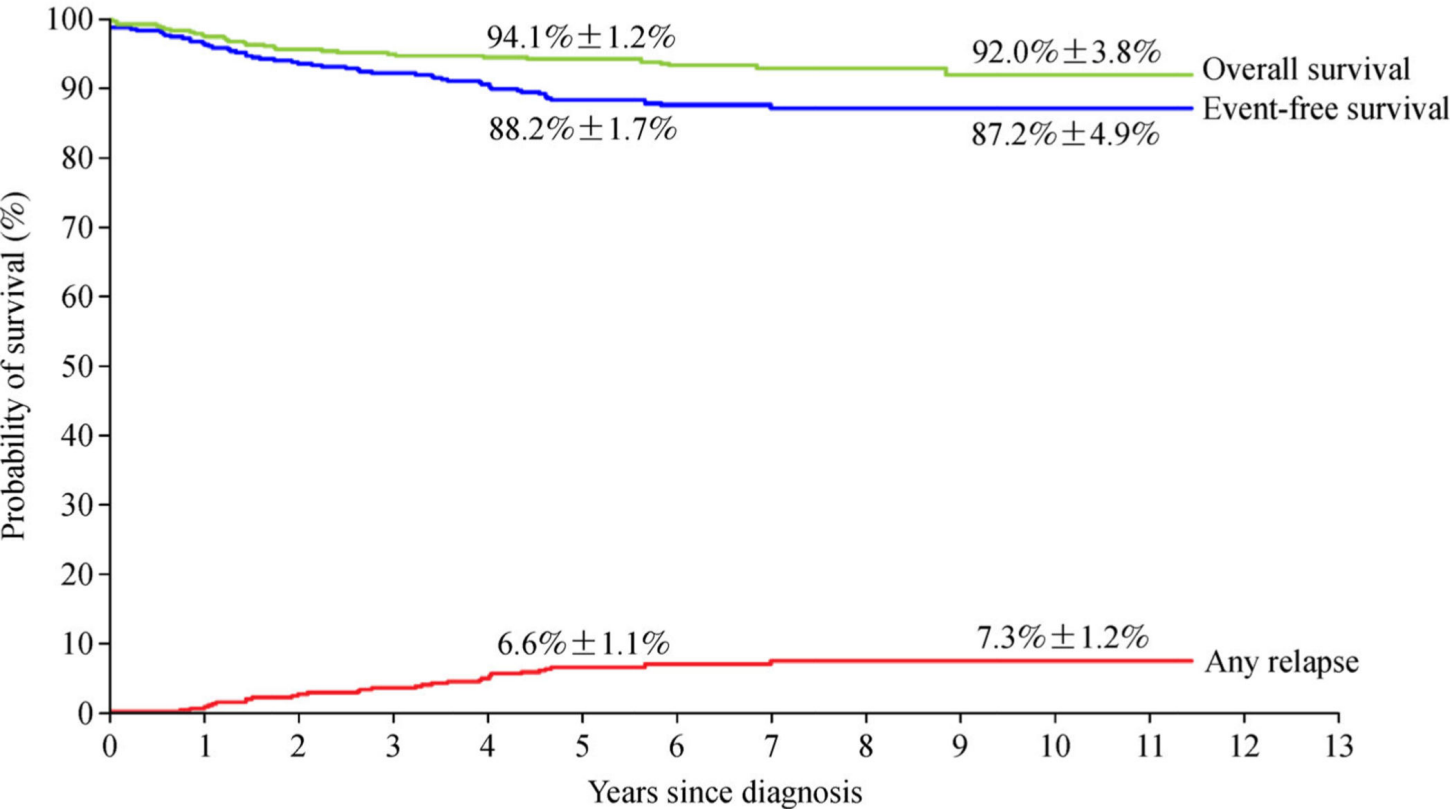
Kaplan–Meier and Kalbfleisch and Prentice analyses of outcomes in 598 children with acute lymphoblastic leukemia. The 5-year and 10-year results are shown on the curves.

Contemporary risk-directed treatment has improved 5-year event-free survival and overall survival rates in childhood acute lymphoblastic leukemia (ALL) to over 80% and 90%, respectively, and has decreased the cumulative risk of relapse to less than 10% in many clinical trials (Table 1) [1–11]. In a recently completed St. Jude Total Therapy Study 16, the 5-year event-free survival rate was 88.2% and the 5-year cumulative risk of any relapse 6.6% among 598 evaluable patients [9] (Fig. 1). Despite the significant reduction of cumulative risk for a CNS relapse or any relapse and a corresponding increase in event-free survival, the overall survival rate (94.1%) in the Study 16 was similar to that (93.5%) in the Study 15 [11]. This outcome suggests that the intensity of conventional chemotherapy has reached its limit of tolerance and can no longer be “pushed” to obtain improved results. Thus, if we intend to boost cure rates and the quality of life of children with ALL in the coming decade, it will be important to replace toxic chemotherapy with carefully selected components of molecular therapeutics and cellular- and immunotherapy, preferably those that lack overlapping toxicity with chemotherapy [12]. This review will focus on the molecular genetic features of the major subtypes of ALL and describe recent advances in targeted therapy that promise to secure improved clinical outcomes.

### Genomic landscape of acute lymphoblastic leukemia

Recent studies have refined the classification of B- and T-lineage ALL into gene expression-based subgroups, and the comprehensive integration of specific mutated genes and pathways for each subgroup has significantly improved our understanding of the disease biology. Cases can be classified based on whole transcriptome sequencing (RNA-seq); aneuploidy or other chromosomal abnormality; deregulation of known transcription factors by mutations or rearrangement; or activation of kinase alterations into at least 23 subtypes of B-ALL and 9 subtypes of T-ALL, many of which have prognostic or therapeutic implications [13–15]. However, heterogeneity still exists in treatment response among patients with the same genetic subtypes due to cooperative mutations, germline genetic variants, and other host or environmental factors [16]. Thus, key genetic alterations will need to be combined with clinical variables and response to therapy (as determined by minimal residual disease (MRD) measurements) to avoid over- or under-treatment. In this regard, the prognostic and therapeutic relevance of current approaches to genetic classification of T-ALL remains tenuous, even in the context of MRD-stratified therapy, so that most T-ALL patients still require intensive chemotherapy for cure [12].

### Reduced dose intensity of treatment for patients with favorable genotypes and excellent early treatment responses

Contemporary protocols allow the reduction of treatment dose intensity to improve the quality of life for low-risk patients while maintaining their cure high rates. In the Associazione Italiana di Ematologia e Oncologia Pediatrica (AIEOP) and Berlin-Frankfurt-Münster (BFM) ALL 2000 protocol, patients 1 to 17 years old with standard-risk B-ALL, defined by the absence of high-risk genetic features (*BCR-ABL1, KMT2A-AFF1*) and the lack MRD disease (i.e., level < 1 × 10^–4^) on days 33 and 78 from the start of remission induction treatment, were randomized to receive standard or reduced delayed intensification treatment [17]. This modification resulted in a poorer overall 8-year disease-free survival (89.2% ± 1.3% vs. 92.3% ± 1.2%) and overall survival (96.1% ± 0.8% and 98.0% ± 0.6%) except for the patients with *ETV6-RUNX1*-positive ALL or ages of 1 to 6 years who fared equally well in both treatment arms. This study shows that treatment reduction in this context is only feasible in specific subgroups of patients with standard-risk ALL.

In St. Jude Total Therapy Study 15, only low-risk B-ALL patients with *ETV6-RUNX1*-postive or hyperdiploid > 50 ALL and negative MRD ( < 1 × 10^–4^) on day 19 of remission induction had a low cumulative risk of relapse (1.9% and 3.8%, respectively) as compared to an unacceptably high cumulative risk of relapse (9.5%) in low-risk (i.e., NCI standard-risk B-ALL) patients with other genotypes and negative MRD on day 19 [18]. In a Children’s Oncology Group study, the 56 patients with NCI standard-risk B-ALL and undetectable MRD by high-throughput sequencing ( < 10^–5^) on day 29 of remission induction had an excellent 5-year event-free survival of 98.1% and an overall survival of 100% [19]. Hence, only two subgroups appear to be suitable candidates for reduced treatment: (1) B-ALL patients with a favorable genotype (*ETV6-RUNX1* positivity or hyperdiploidy > 50) who achieve an early negative MRD status (10^–4^) by conventional methods or (2) other NCI standard-risk B-ALL patients with negative MRD by high-throughput sequencing ( < 10^–5^) at the end of remission induction.

Two other newly identified genotypes of B-ALL, *DUX4*-rearranged (with overexpression of *DUX4* and transcriptional deregulation of ERG) and ETV6-RUNX1like (with gene expression profile similar to that of *ETV6-RUNX1*-positive ALL and coexisting *ETV6* and *IKZF1* alterations), appeared to have favorable prognosis in retrospect studies [14,20,21]. However, because small numbers of patients were studied, their favorable prognosis required confirmation. At present, there are no reliable biomarkers that could be used to identify subsets of T-ALL patients who might benefit reduced-intensity chemotherapy [9,12,22].

### High-risk genetic subtypes that benefit from targeted therapy

#### Philadelphia chromosome-positive ALL

Although addition of imatinib, the first-generation ABL1 tyrosine kinase inhibitor, to conventional treatment has improved outcome in children with Philadelphia chromosome (*BCR-ABL1*)-positive ALL [23–25], refractory or relapsed disease remains a difficult problem in these cases. To overcome resistance-inducing ABL1 kinase domain mutations, dasatinib and nilotinib, two second-generation tyrosine kinase inhibitors, were developed [26]. Dasatinib, the more commonly used dual ABL and SRC kinase inhibitor, can cross the blood-brain barrier [27].

Two nonrandomized clinical trials suggested that dasatinib (at 60 mg/m^2^ per day) can secure results comparable to those achieved with imatinib, with a lower proportion of dasatinib-treated patients undergoing allogeneic hematopoietic cell transplantation or cranial irradiation than imatinib-treated patients [28,29]. However, because of the use of historical controls and the differences in the proportion of patients undergoing transplantation and cranial irradiation in imatinib- vs. dasatinib-treated patients, the relative efficacy of these two agents remains uncertain. Moreover, despite transplantation in 32% and 14% of the patients and prophylactic cranial irradiation for patients with a CNS3 status, 4 of 60 and 4 of 106 dasatinib-treated patients developed CNS relapse in the two studies, respectively [28,29].

In St. Jude Total Therapy Study 16, the 15 Philadelphia chromosome-positive ALL patients treated with dasatinib (80 mg/m^2^ per day) had an excellent 5-year event-free survival rate of 71% and none developed CNS relapse, despite total omission of cranial irradiation and transplantation limited to 1 patient [9]. The Chinese Children’s Cancer Group recently conducted the first randomized study comparing the efficacy of imatinib (300 mg/m^2^ per day) with that of dasatinib (80 mg/m^2^ per day) in children with Philadelphia chromosome-positive ALL [30]. By the study design, no patients received prophylactic cranial irradiation and only 2% of the patients with MRD ≥ 1% after remission induction received allogeneic transplantation. The dasatinib-treated patients had a significantly better event-free survival and overall survival than the imatinib-treated patients and only one of 92 dasatinib-treated patients developed CNS relapse. These findings suggest that dasatinib administered at 80 mg/m^2^ per day improved outcome by achieving optimal therapeutic level both systemically and in the CNS.

Ponatinib is the most potent third-generation of ABL1 class tyrosine kinase inhibitors and is active against cases with mutated *ABL1*, including Thr315Ile [26]. A recent adult study incorporating this drug achieved an excellent 3year event-free survival of 70%, with only 20% of the patients undergoing allogeneic transplantation [31]. Investigation of this drug in the pediatric population is warranted.

#### Philadelphia chromosome-like ALL

Philadelphia chromosome-like B-ALL, characterized by an activated kinase gene expression profile resembling that of Philadelphia chromosome-positive ALL with a high frequency of IKZF1 alterations but lacking *BCR-ABL1* fusion, occurs in approximately 12% of childhood B-ALL cases [32]. However, this genotype is heterogeneous and has a wide range of genetic alterations, many of which respond to different tyrosine kinase or signal pathway inhibitors [32–34]. Approximately half of these patients have *CRLF2* (cytokine receptor-like factor 2) rearrangements, leading to activation of PI3K/AKT/mTOR and JAK-STAT signaling, especially in older patients and in Native American and Hispanic or Latino populations [35]. Among childhood and adolescent patients with *CRLF2* rearrangement, approximately half have concomitant *JAK2* or *JAK1* mutations, which may respond to JAK-STAT inhibitors such as ruxolitinib [32–35].

Among the other half of patients with Philadelphia chromosome-like ALL lacking *CRLF2* rearrangements, 15%–20% have rearrangements in *ABL1, ABL2*, *CSF1R*, or platelet-derived growth factor receptor (*PDGFR*) α or β and would likely respond to ABL-class tyrosine kinase inhibitors. Another 10%–15% of patients have lesions that activate JAK–STAT signaling, including *JAK2* fusions or truncating rearrangements in erythropoietin receptor [32–35]. There are other uncommon kinase fusion events involving *NTRK3, PTK2B, TYK2, FLT3, FGFR1*, and BLNK, which have been responsive to TRK inhibitor, FAK inhibitor, TYK2 inhibitor, FLT3 inhibitor, sorafenib/dasatinib, and SYK/MEKi, respectively, in preclinical settings [32,33].

Although a high proportion of Philadelphia chromosome-like ALL cases have an unfavorable outcome, approximately 40% of the childhood cases are highly curable even with low-intensity chemotherapy. This subgroup can be readily identified with negative MRD status upon completion of remission induction [36]. Hence, it is mandatory to measure MRD levels to avoid overtreatment of these cases.

#### Hypodiploid ALL

Hypodiploid ALL, found in 2% to 3% of childhood ALL cases, is also a heterogeneous disease, comprising several subgroups with different biologic and prognostic features [37]. Near-haploid ALL (25–29 chromosomes) is characterized by genetic alterations affecting *RAS* signaling and receptor tyrosine kinase signaling, and a high frequency of *IKZF3* alterations [37]. Low-hypodiploid cases (33–39 chromosomes) often have alterations in *TP53, IKZF2*, and *RB1*. Alterations in *TP53* have been identified in as many as 90% of patients with low hypodiploid ALL, with approximately 50% of these cases having germline *TP53* alterations [37], which are associated with inferior event-free survival and overall survival as well as an increased risk of developing secondary cancer [38], regardless of ploidy status. Thus, all patients with low hypodiploid ALL should be tested for a germline *TP53* pathogenic variant (e.g., Li-Fraumeni syndrome, a well-known hereditary cancer predisposition syndrome [39]).

A recent multinational study of 306 cases further characterized the clinical and biologic prognostic hallmarks of hypodiploid ALL [40]. The results showed that despite contemporary treatment, patients with hypodiploid ALL continue to have poor overall outcome with an 8-year survival rate for the entire cohort of only 57.5%. It also demonstrated that hypodiploidy may accompany specific driver genetic abnormalities with known prognostic significance, such as *BCR-ABL1, TCF3-PBX1, ETV6-RUNX1*, and *KMT2A* rearrangements. These cases should be treated according to their driver mutations and MRD level after remission induction because the treatment outcomes closely reflect each specific driver mutation [40]. In this regard, only one of 18 hypodiploid patients with concomitant *ETV6-RUNX1* relapsed, while the remaining 17 patients were alive in long-term remission. Three independent favorable prognostic factors were identified: (1) negative MRD at the end of remission induction, (2) high hypodiploidy with 44 chromosomes, and (3) treatment in MRD-stratified protocols. Importantly, allogeneic transplantation failed to improve outcome compared with chemotherapy alone, especially for patients who achieved a negative MRD status after remission induction, a finding confirmed by a Children’s Oncology Group study that treated patients during the same time period [41]. A recent preclinical study identified Bcl-2 as a key therapeutic target and demonstrated the efficacy of a selective Bcl-2 inhibitor, venetoclax, in hypodiploid ALL, providing a promising treatment strategy to improve outcome in this disease [42].

### Other genotypes that may be targetable by venetoclax

Deregulated cell death pathways contribute to treatment failure in many cancers, including certain subtypes of ALL. Intrinsic apoptotic signaling is regulated by proapoptotic BCL-2 homology domain 3 (BH3) proteins that trigger apoptotic cell death and by antiapoptotic molecules including BCL-2 that counter-regulate apoptosis induction. A treatment strategy of inhibiting antiapoptotic regulators led to the development of the BCL-2 inhibitor venetoclax. A functional assay, BH3 profiling, measured the state of the mitochondrial apoptosis pathway in cells, and was developed to predict types of cancers that would respond to this class of drugs [43]. BH3 profiling and other preclinical methods have identified a number of high-risk leukemias, including early T cell precursor ALL [44], immature T-ALL [45], *KMT2A* (*MLL*)-rearranged ALL [46,47] and Philadelphia chromosome-positive ALL [48,49] as well as *TCF-HLF*-positive ALL, the most aggressive form of ALL [50], all of which are Bcl-2 dependent and sensitive *in vitro* and *in vivo* to treatment with venetoclax. Clinical trials are warranted to determine if venetoclax can improve outcome in these high-risk subtypes of leukemia.

#### *MEF2D*-rearranged ALL

Rearrangements between *MEF2D* (myocyte enhancer factor 2D) and various genes (*BCL9, CSF1R, DAZAP1, HNRNPUL1, HNRNPH1*, SS18, FOXJ2) occurred in approximately 2% to 3.5% of patients with a cytoplasmic μ chain pre-B immunophenotype, older presenting age (median 12 years) and poor outcome (5-year survival ranging between 30% to 70%) [14,51,52]. In a study of relapsed ALL, *MEF2D-BCL9* fusion was found in 4 of 59 relapsed or refractory ALL patients who had older age (10 to 13 years), very early relapse (8 to 15 months from diagnosis), and very poor outcome (0% survival) [53]. The rearrangements resulted in upregulation of pre-B cell receptor signaling molecules, downregulation of JAK-STAT signaling pathway, enhanced MEF2D transcriptional activity and activation of HDAC9 expression, with sensitivity to histone deacetylase inhibitors such as panobinostat [14,51,52]. Studies are needed to assess the heterogeneity of treatment responses among patients with different fusion partners and whether these cases respond to treatment with histone deacetylase inhibitors.

#### *PAX5*-driven B-ALL

Recent integrated genomic analyses identified two subtypes of B-ALL with frequent alterations of the Blymphoid transcription factor *PAX5* [13,54]. One, designated *PAX5alt*, has diverse alterations (mutations, intragenic amplifications or rearrangement) of the gene, while the other, *PAX5P80R*, has *PAXp.Pro80Arg* and biallelic *PAX5* alterations. In one study, patients with either set of changes had a higher median age (22 and 15.4 years vs.13 years for the total patient cohort) and a lower MRD level at the end of induction (7.2% and 29.4% vs.37.8% for the total cohort). Not surprisingly, pediatric patients had intermediate treatment outcomes: 5-year event-free survival rates of 75% ± 14.2% and 71.5% ± 7%, respectively [13]. To date, no molecularly targeted therapy has been identified.

### Mixed-lineage acute leukemias

Recent comprehensive genomic and immunophenotypic analyses have provided important insights into the biology and treatment response among immunophenotypically defined subtypes of acute leukemia expressing both lymphoid and myeloid markers, which account for 2% to 3% of childhood acute leukemias [55,56]. These so-called mixed-phenotype acute leukemias can be broadly classified into B-myeloid and T-myeloid subtypes. *KMT2A*-rearranged, Philadelphia chromosome-positive and *ZNF384*-rearranged leukemias are the most common genotypes among B-myeloid leukemia, and biallelic WT1 alterations are common in the T-myeloid subtype, which shares genomic features such as *RAS* and *JAK–STAT* pathway mutations with early T cell precursor ALL [55]. Overall, B-myeloid and T-myeloid leukemias have similar treatment outcomes except for *KMT2A*-rearranged cases, which typically have a very poor prognosis [47]. In a retrospective multinational study, lymphoid-directed therapy was superior to myeloid-directed therapy for most pediatric patients with mixed-phenotype acute leukemias, except for a minority of patients with CD19-negative leukemia, who benefitted from myeloid-directed therapy [56].

### Leukemias without available molecular targeted therapy

A substantial proportion of high-risk ALL patients with poor early treatment responses, for example, those with iAMP21 [57], do not have targetable genetic lesions or effective molecular therapeutics available. Current treatment approaches besides intensive chemotherapy that offer hope for this subgroup are immunotherapy and adoptive cell therapy. Blinatumomab, a bispecific T cell engager antibody, by binding to CD3 on the surface of T cells and CD19 on leukemia cells, initiates T cell receptor-mediated activation and killing of CD19-positive B-ALL [58]. It has been shown to improve outcome in multiple studies of adults with refractory, relapsed and newly diagnosed Philadelphia chromosome-negative or positive ALL [58]. In fact, the combination of ABL tyrosine kinase inhibitor and blinatumomab may synergistically improve the outcome of patients with Philadelphia chromosomepositive ALL [59]. Blinatumomab was recently approved for pediatric patients with relapsed or refractory ALL after a phase I/II trial showing 39% complete remission rate with 52% of the responders achieving an MRD negative status within the first two cycles of treatment [60]. While blinatumomab is generally well-tolerated, it has been associated with severe and potentially life-threatening adverse events, including cytokine release syndrome and neurotoxicity, which can occur simultaneously or independently [61]. The rate of cytokine release syndrome could be reduced by a debulking sequential combination approach [58]. An ongoing adult study is investigating the combination of immune checkpoint blockade with blina-tumomab treatment to enhance T cell activation and hence augment the activity of the antibody (NCT03160079) [59].

The anti-CD22/calicheamicin conjugate (inotuzumab ozogamicin) is an effective FDA-approved agent in the treatment of adults with relapsed ALL [62]. Among 51 children with relapsed or refractory ALL treated with inotuzumab ozogamicin in a compassionate-use program, complete remission was achieved in 67% of the patients with overt marrow disease, and the majority (71%) of the responders were negative for MRD [63]. The treatment was well tolerated; sinusoidal obstruction syndrome was not observed in any patients during treatment but developed in 11 of 21 patients (52%) after hematopoietic cell transplantation. The combination of non-intensive chemotherapy and blinatumomab plus inotuzumab have been evaluated in adults with ALL in first relapse with encouraging results [64]; similar studies have yet to be conducted in children.

The most effective FDA-approved cellular therapy is the use of CD19-specific chimeric antigen receptor (CAR) T cells containing a 4–1BB (CD137) domain to provide a costimulatory signal. In a recent global study of 75 patients with relapsed or refractory B-ALL treated with tisagenlecleucel (CD19-targeted CAR T cells), the overall complete remission rate within 3 months was 81%, and the 12-month event-free and overall survival rates were 50% and 76%, respectively [65]. The cytokine release syndrome occurred within a median time to onset of 3 days (range, 1 to 22) in 77% of the patients, of whom 47% were admitted to intensive care unit and 48% of whom required tocilizumab. Neurologic adverse events (encephalopathy, confusion, delirium, tremor, agitation, somnolence and seizure) occurred in 40% of the patients within 8 weeks after infusion. Of the 22 relapsed cases, 1 had CD19^+^ recurrence, 15 had CD19^–^ recurrence (3 with concomitant CD19^+^ blasts) and 6 had an unknown CD19 status. Interestingly, CAR-T cells can eradicate leukemia cells in central nervous system and testes [66,67], sparing patients with extramedullary disease from receiving local irradiation.

In a recent report of a phase 1 trial testing a CD22targeted CAR-T cell therapy in 21 children and adults, including 17 who were previously treated with CD19-directed immunotherapy, dose-dependent antileukemic activity was observed, with complete remission achieved in 11 of the 15 patients receiving ≥ 1 ± 10^6^ CD22-CART cells per kg of body weight, including all 5 patients with CD19^dim^ or CD19^–^ B-ALL [68]. However, the median remission duration was only 6 months. Relapses were associated with a diminished CD22 site density that likely permitted CD22^+^ cell escape from killing by the CD22-CAR-T cells. Thus, antigen escape is the primary cause of relapse in the majority of patients treated with either CD19- or CD22-targeted CAR-T cell therapy. These findings strongly support current research to develop simultaneous CD19- and CD22-targeting with CAR-T cells to disallow the opportunity and time for sequential loss of both antigens [69]. Although immunotherapy and adoptive cellular therapy have been used in the relapse or refractory setting, they are being brought forward to newly diagnosed B-ALL patients and promise to improve outcome of these patients.

For T-ALL patients, curative therapeutic options for relapsed or refractory disease beyond allogeneic transplantation are lacking. Translating CAR-T cell therapies into the setting of T-ALL have not yet been successful, and there is a theoretical risk of so-called fratricide by T cell-targeted clones because of the shared expression of target antigens between CAR-T cells and T-leukemia cells, and the risk of severe life-threatening immunodeficiency from elimination of normal T lymphocytes [70,71]. Recently, fratricide-resistant CD7, CD5, and CD1a-targeted CAR-T cells with specific cytotoxicity *in vitro* and antileukemic activity *in vivo* in xenograft models [72,73], and universal allo-tolerant off-the-shelf CAR-T cells generated by genomic editing [74,75] have been developed in an attempt to overcome those limitations but have yet to be tested in a clinical setting.

Nelarabine is the only drug approved specifically for relapsed T-ALL. In a recent Children’s Oncology Group trial (AALL0434) for T-ALL, in which all intermediate- or high-risk patients received cranial irradiation, those randomized to receive nelarabine had an excellent treatment outcome [76]. The 4-year disease-free survival was 88.9% for patients who received nelarabine, compared with 83.3% for those treated without this agent. However, this improvement was noted only in the subset of patients randomized to receive high-dose methotrexate and not in the other subset randomized to receive escalating doses of methotrexate. Thus, additional studies are needed to determine the true efficacy of nelarabine. Among several antibodies being evaluated, the anti-CD38 monoclonal antibody daratumumab appears promising, as this target is overexpressed on T-ALL cells but expressed in very low levels on normal lymphoid and myeloid cells, and the antibody was very effective against T-ALL in human xenograft models [77].

## Conclusions

With improved genomic sequencing and the development of novel molecular, immunological and cellular therapy, we are now entering an exciting era of precision medicine for ALL. Replacing toxic chemotherapy with precisely targeted therapy promises to improve not only the cure rate of this disease, but also the quality of life of patients. Table 2 summarizes some of the potential therapeutic intervention for various subtypes of ALL. Similar to conventional chemotherapy, we should focus on rational combinations of targeted therapies with non-overlapping toxicities, allowing the agents to act synergistically to kill leukemia cells while sparing normal tissues from excessive toxicity. For example, while venetoclax by itself has limited activity against T-ALL, with the exception of early Tcell precursor or immature T cell ALL [44,45], the combination of venetoclax and navitoclax, a Bcl-2 inhibitor that also inhibits Bcl-X_L_ and Bcl-w, may achieve effective activity against T-ALL while sparing patients from profound navitoclax-induced thrombocytopenia [78]. This hypothesis is being tested in a study (NCT3181126) for children ≥ 4 years old and adults with refractory or relapsed ALL.

## Figures and Tables

**Table 1:** Treatment results from selected clinical trials

Study group	Years of study	No. of patients	Age range (year)	T cell ALL (%)	5-year cumulative rate of any relapse (%)	5-year EFS (%)	5-year survival (%)	Data source

AIEOP-BFM 2000	2000–2006	4839	1–17	13.2	13.2	81.4±0.6	91.9±0.4	Möricke *et al*. (2016) [1]
CoALL-07-03	2003–2010	743	1–18	12.9	NA	83±0.3	NA	Escherich *et al*. (2013) [2]
COG	2000–2005	7153	0–22	7	7.2	NA	90.4±0.5	Hunger *et al*. (2012) [3]
DCOG-10	2004–2011	778	1–18	14.2	8.3	87.0±1.2	91.9±1.0	Pieters *et al*. (2016) [4]
DFCI 05-001*	2005–2010	697	1–18	0	9.0	86±3	92±2	Vrooman *et al*. (2018) [5]
EORTC 58951	1998–2008	1947	1–18	15.2	14.7	82.6±0.9	89.7±0.7	Domenech *et al*. (2014) [6]
MRC UKALL 2003	2003–2011	3126	1–25	12	8.8	87.3±1.4	91.6±1.2	Vora *et al*. (2014) [7]
NOPHO-2008	2008–2014	1022	1–9	9.1	13	89±1	94±1	Toft *et al*. (2018) [8]
NOPHO-2008	2008–2014	266	10–17	25.2	7.0	80±3	87±2	Toft *et al*. (2018) [8]
SJCRH 16	2000–2017	598	0–18	17.4	6.6	88.2±3.3	94.1±2.4	Jeha *et al*. (2019) [9]
TPOG	1999–2010	152	0–18	7.2	NA	84.2±3.0	90.2±2.4	Liu *et al*. (2014) [10]

Abbreviations: ALL, acute lymphoblastic leukemia; AIEOP, Associazione Italiana di Ematologia Pediatrica Group; BFM, Berlin-Frankfurt-Münster; CoALL, Cooperative ALL Study Group; COG, Children’s Oncology Group; DCOG, Dutch Children’s Oncology Group; DFCI, Dana-Farber Cancer Institute consortium; EFS, event-free survival; EORTC–CLG, European Organisation for Research and Treatment of Cancer–Children Leukemia Group; MRC UKALL, Medical Research Council UK acute lymphoblastic leukemia; NA, not available; NOPHO, Nordic Society of Pediatric Hematology and Oncology; SJCRH, St. Jude Children’s Research Hospital; TPOG, Taiwan Pediatric Oncology Group.

* T-ALL patients not included.

**Table 2:** Clinical implications and potential therapeutic implications of selected subtypes of ALL

Subtype	Risk group	Therapeutic approach

*ETV6-RUNX1*	Low	Reduced dose intensity if MRD <10^–4^ during early induction or <10^–5^ MRD at the end of induction
High-hyperdiploid	Low	Reduced dosed intensity if MRD <10^–4^ during early induction or <10^–5^ MRD at the end of induction
*DUX4*-rearranged	Low	Standard dose intensity, MRD-adapted
*ETV6-RUNX1*-like	Standard	Standard dose intensity, MRD-adapted
*TCF3-PBX1*	Standard	Standard dose intensity, MRD-adapted, high-dose methotrexate
*PAX5 P80R*	Intermediate	Standard dose intensity, MRD-adapted
*PAXalt*	Intermediate	Standard dose intensity, MRD-adapted
*ZNF384*-rearranged	Intermediate	Standard dose intensity, MRD-adapted
Philadelphia chromosome-positive	High	ABL tyrosine kinase inhibitors, retinoids, Bcl-2 inhibitors, FAK inhibitors
Philadelphia chromosome-like	Variable	Second or third generation ABL tyrosine kinase inhibitors, JAK inhibitors, Bcl-2 inhibitors
Hypodiploid	High	Intensive dose intensity, MRD-adapted, Bcl-2 inhibitors
*KMT2A*-rearranged	High	DOTL1i, Menin inhibitors, Bcl-2 inhibitors
*TCF-HLF*	High	Intensive dose intensity, Bcl-2 inhibitors
*MEF2D*-rearranged	High	Histone deacetylase inhibitors, bortezomib
Early T cell precursor	High	Intensive dose intensity, Bcl-2 inhibitors
